# An International Environmental Scan of Social Housing for Older Adults

**DOI:** 10.1093/geroni/igaa057.3347

**Published:** 2020-12-16

**Authors:** Sander Hitzig, Christine Sheppard, Ariana Holt, Andrea Austen, Miller Glenn

**Affiliations:** 1 Sunnybrook Hospital, North York, Ontario, Canada; 2 Sunnybrook Research Institute, Toronto, Ontario, Canada; 3 Canadian Urban Institute, Toronto, Ontario, Canada; 4 City of Toronto, Toronto, Ontario, Canada; 5 Canadian Urban Institute, Toronto, Canada

## Abstract

The City of Toronto is creating a standalone housing corporation to focus on the specific needs of low-income older adults living in social housing. A key focus of this new corporation will be to provide housing, health and community support services needed to optimize older adult tenants’ ability to maintain their tenancy and age in place with dignity and in comfort. To support this work, we conducted an environmental scan of service delivery models that connect low-income older adults living in social housing with health and support services. Desktop research was undertaken to identify relevant programs. For each model, key details were extracted including housing type, services offered, provider information, rent structure and funding sources. The scan identified 34 examples of social housing programs for older adults run by public, private and non-profit agencies across Canada, the United States and Europe that integrated health and supportive services. Successful models were those that understood the needs of tenants and developed collaborative partnerships with health and social service providers to create flexible place-based programs. A common challenge across jurisdictions was privacy legislation that made it difficult to share health and tenancy data with program partners. The presence of on-site staff that focused on building trust and community among tenants was considered key for identifying tenants requiring additional supports in order to age in place. These insights offer important considerations on how integrated supportive housing service models promote housing stability and support better health and wellbeing among older adults residing in social housing.

